# 阿法替尼一线治疗5例晚期肺腺癌患者的不良反应分析及相关文献回顾

**DOI:** 10.3779/j.issn.1009-3419.2014.04.09

**Published:** 2014-04-20

**Authors:** 虹 陶, 丽丽 郭, 俊舫 唐, 允中 朱, 丽艳 徐, 弃逸 孟, 玮 武, 明智 李, 卫华 吴, 丽 仝, 洪波 吴, 亮 史, 喆 刘

**Affiliations:** 101149 北京，首都医科大学附属北京胸科医院肿瘤内科 Department of Medical Oncology, Beijing Chest Hospital, Capital Medical University, Beijing 101149, China

**Keywords:** 阿法替尼, 表皮生长因子受体, 酪氨酸激酶抑制剂, 肺肿瘤, 不良反应, Afatinib, Epidermal growth factor receptor, Tyrosine kinase inhibitor, Lung neoplasms, Adverse event

## Abstract

**背景与目的:**

阿法替尼是一种新型的低分子量人类表皮生长因子受体（human epidermal growth factor receptor, HER）家族抑制剂，它属于第二代表皮生长因子受体-酪氨酸激酶抑制剂（epidermal growth factor receptor-tyrosine kinase inhibitors, EGFR-TKIs），在临床前和临床研究中显示了该药对具有表皮生长因子受体（epidermal growth factor receptor, EGFR）活性突变的非小细胞肺癌（non-small cell lung cancer, NSCLC）的疗效。本研究关注阿法替尼治疗晚期肺腺癌患者的安全性。

**方法:**

确诊为Ⅲb期或Ⅳ期肺腺癌、具有*EGFR*突变的患者，一线给予阿法替尼每日40 mg口服，直至疾病进展。观察不良反应、疗效及生存情况。

**结果:**

最常见不良反应为腹泻（*n*=5, 100%）、皮疹（*n*=4, 80%）、粘膜炎/口腔炎（*n*=4, 80%）。总体不良反应程度较轻，均≤Ⅲ级，相对最严重的副反应为粘膜炎/口腔炎。腹泻虽发生于所有患者，但程度较轻。共有3例患者因不良反应暂停药、减量。在4例可评价疗效的患者中，部分缓解（partial response, PR）2例（50%），疾病稳定（stable disease, SD）1例（25%），疾病进展（progressive disease, PD）1例（25%）。中位无进展生存期（progression-free survival, PFS）9.7个月，中位总生存期（overall survival, OS）18.4个月。

**结论:**

阿法替尼一线治疗晚期肺腺癌患者疗效确切，常见不良反应除腹泻、皮疹外，还应关注粘膜炎/口腔炎的发生。由于本研究入组人数较少，此结论尚需得到研究者的进一步关注。

## Abstract (en)

**Background and objective:**

Afatinib is an irreversible ErbB-family blocker with a clinical activity in non-small cell lung cancer with epidermal growth factor receptor (EGFR) mutations. The aim of this study is to assess the safety of afatinib in patients with advanced lung adenocarcinoma.

**Methods:**

Patients with lung adenocarcinoma (stage Ⅲb or Ⅳ) with *EGFR* mutations were first-line treated with an oral administration of afatinib (40 mg/d) until disease progression. Adverse events, effects, and survival condition were observed.

**Results:**

The most common adverse events were diarrhea (*n*=5, 100%), skin rash (*n*=4, 80%), and mucositis/stomatitis (*n*=4, 80%). Moderate toxicities not exceeding grade 3 were observed. Relatively, the most serious adverse reaction was mucositis/stomatitis. Mild diarrhea occurred in all patients. Three patients experienced temporary drug withdrawal and dose reduction because of adverse reaction. Among the four patients who were evaluated, partial response was observed in two patients (50%), one with stable disease (25%) and one with progressive disease (25%). Median progression-free survival was 9.7 months, whereas median overall survival was 18.4 months.

**Conclusion:**

Afatinib was approved as first-line treatment for patients with advanced lung adenocarcinoma. The most common adverse events were diarrhea and skin rash. However, mucositis/stomatitis related to afatinib should also be considered. Considering the small number of cases, the conclusion requires more trials for confirmation.

化疗作为肿瘤治疗的主要手段之一，在晚期肺癌综合治疗中具有举足轻重的地位。肺癌中约85%为非小细胞肺癌（non-small cell lung cancer, NSCLC），化疗对NSCLC的有效率不足40%^[1]^，其疗效已达到瓶颈。研究^[2-4]^证实，作用于EGFR通路的第一代表皮生长因子受体酪氨酸激酶抑制剂（epidermal growth factor receptor tyrosine kinase inhibitors, EGFR-TKIs）吉非替尼、厄洛替尼对具有EGFR活性突变的NSCLC疗效确切，且副反应较小。本研究选用第二代EGFR-TKIs阿法替尼（Afatinib, Tomtovok, Tovok, BIBW 2992），观察其对晚期初治肺腺癌患者的不良反应及疗效。

## 1 对象与方法

### 1.1 对象

2010年11月-2012年9月于北京胸科医院肿瘤内科住院的患者，符合勃林格殷格翰公司LUX-Lung 6研究的入组条件（本研究是LUX-Lung 6研究的一部分）。要求：经病理证实的Ⅲb期（胸腔积液或心包积液）或Ⅳ期肺腺癌初治患者；肿瘤活检组织检测到*EGFR*突变（19外显子缺失；L858R；20外显子3插入；L861Q；G719S，G719A，G719C；T790M；S768I）；具有可测量的病灶；年龄≥18岁；ECOG PS评分0分或1分；预期寿命至少3个月；签署知情同意书。入选患者共5例（表 1），其中男性3例，女性2例，年龄范围48岁-63岁，中位年龄59岁。吸烟/曾吸烟者3例，从不吸烟者2例。肿瘤分期均为Ⅳ期，ECOG PS评分0分者1例，1分者4例。入组患者突变情况：1例19外显子缺失，3例L858R，1例既有19外显子缺失又有L861Q突变。

**1:** 患者特征、疗效及生存情况 Characteristics, effect and survival conditions of all patients

	Patient 1	Patient 2	Patient 3	Patient 4	Patient 5
Gender	Male	Female	Female	Male	Male
Age (yr)	56	62	63	59	48
Smoking status	Smoking	Non-smoking	Non-smoking	Ex-smoking	Smoking
Stage	Ⅳ	Ⅳ	Ⅳ	Ⅳ	Ⅳ
ECOG PS	1	1	0	1	1
Type of EGFR mutation	Exon 19 deletion, L861Q	L858R	L858R	Exon 19 deletion	L858R
The best response	PR	PR	NK^#^	SD	PD
PFS (month)	NK	9.7	NK	18.1	1.1
OS (month)	-- ^*^	18.4	-- ^**^	> 29.5	3.4
ECOG: Eastern Cooperative Oncology Group; PS: performance status; PR: partial response; NK: not know; SD: stable disease; PD: progressive disease; PFS: progression-free survival; OS: overall survival; #: chest X ray showed lesion decreased; ^*^: withdraw the trial because of other reason; ^**^: withdraw the trial because of mucositis/stomatitis.					

### 1.2 治疗方案

每日口服阿法替尼单药40 mg；如无严重不良事件，剂量可加至50 mg；如出现一定级别的不良事件，可减量至30 mg。服药至疾病进展。阿法替尼由勃林格殷格翰公司提供。

### 1.3 不良反应

根据CTC 3.0毒副反应标准进行评价。

### 1.4 近期疗效

根据RECIST 1.1疗效评价标准进行评价。每6周进行一次肿瘤评估，48周后每12周进行一次评估。

### 1.5 随访和生存

疾病进展后每2个月随访生存情况。无进展生存期（progression-free survival, PFS）定义为：自随机开始到肿瘤进展或受试者死亡的时间（以先出现的为准）。总生存期（overall survival, OS）定义为：自随机开始到受试者死亡的时间。

## 2 结果

### 2.1 不良反应

#### 2.1.1 种类及比例

最常见的药物相关不良反应（表 2）为腹泻5例（100%），其次为皮疹4例（80%）、粘膜炎/口腔炎4例（80%），手足皮肤反应、指尖皮肤裂隙、咽部溃疡、甲沟炎、肠梗阻、面部水肿、疲乏、恶心、呕吐、心悸、发热各1例（各20%）。

**2:** 阿法替尼药物相关不良反应 Drug related adverse events of afatinib

Adverse events	Grade 1	Grade 2	Grade 3	Total	%
Gastrointestinal disorders					
Diarrhea	5	0	0	5	100
Nausea	0	1	0	1	20
Vomitting	0	1	0	1	20
Intestinal obstruction	0	1	0	1	20
Skin and subcutaneous tissue disotders					
Rash	3	1	0	4	80
Hand-foot skin reaction	0	0	1	1	20
Fingertip skin cracks	1	0	0	1	20
Administration site conditions and general disorders					
Mucositis/stomatitis	1	0	3	4	80
Pharyngeal ulcer	1	0	0	1	20
Fatigue	1	0	0	1	20
Fever	1	0	0	1	20
Others					
Paronychia	0	1	0	1	20
Facial edema	1	0	0	1	20
Palpitation	1	0	0	1	20

#### 2.1.2 程度

患者发生不良事件的程度较轻，所有患者的不良反应程度均≤Ⅲ级，其中2例患者的不良反应程度≤Ⅱ级。不良事件最严重的程度为Ⅲ级，分别为粘膜炎/口腔炎3例（60%），手足皮肤反应1例（20%）。未见到Ⅳ级-Ⅴ级不良反应及严重不良事件（serious adverse event, SAE）。在各种不良事件中粘膜炎/口腔炎最严重（4例粘膜炎中3例为Ⅲ级）。腹泻虽然发生于所有5例患者中，但程度较轻，均为Ⅰ级，可耐受。

#### 2.1.3 分布特点

不良反应主要集中在胃肠道、皮肤和皮下组织、给药部位异常与全身不适（表 1），共11种，占总体不良事件（共14种）的79%。

#### 2.1.4 对用药的影响

共有3例（60%）停药或减量：2例患者因Ⅲ级粘膜炎/口腔炎暂停药、减量，其中1例因粘膜炎/口腔炎退出研究；1例患者因Ⅲ级手足皮肤反应及Ⅲ级粘膜炎/口腔炎2次暂停药、减量。

### 2.2 近期疗效（表 1）

4例患者可评价疗效，其中完全缓解（complete remission, CR）0例，PR 2例（50%），客观缓解率（objective response rate, ORR）2例（50%），SD 1例（25%），PD 1例（25%）。1例因退出研究未评价疗效，退出研究前复查胸部X线检查提示肺部病灶明显缩小。

### 2.3 随访和生存（表 1）

截至2013年8月，共随访33个月。4例患者去世，1例仍存活。3例获得PFS时间，分别为1.1个月、9.7个月、18.4个月，另2例因退出无法获得，中位PFS 9.7个月。2例获得OS时间，分别为3.4个月、18.4个月，1例仍存活，生存时间大于29.5个月，另2例因退出不参与统计，中位OS 18.4个月。

## 3 讨论

以吉非替尼、厄洛替尼为代表的第一代EGFR-TKIs为小分子物质，特异性作用于EGFR，对其具有可逆的抑制作用。研究表明，吉非替尼、厄洛替尼最常见的副反应包括：皮疹、腹泻、乏力、厌食、消瘦、口腔炎、口腔溃疡等^[3, 4]^，程度较轻，患者耐受良好。然而，获得性耐药问题限制了其使用^[4]^，研究者开始把目光投向第二代EGFR-TKIs。

阿法替尼是一种新型的苯胺-喹唑啉衍生物，具有高度选择性，是低分子量HER家族抑制剂。它属于第二代EGFR-TKIs，能够与EGFR/HER-1、HER-2和HER-4不可逆性结合，抑制受体形成二聚体，加强对下游信号的阻断作用，进一步抑制肿瘤的增殖、转移，从而能够更加有效地控制肿瘤、并且有助于克服耐药^[2, 5]^。

回顾阿法替尼系列临床研究^[6]^，Yap^[7]^报告的Ⅰ期试验入组53例，所患疾病包括肺癌、结直肠癌、食管癌等多种实体瘤，根据剂量从10 mg-50 mg不等分为5组，最常见不良反应为皮疹（68%）、腹泻（64%）；LUX-Lung 1研究^[8]^中390例进入阿法替尼组，起始剂量为50 mg，最常见的不良反应同样为腹泻（87%）、皮疹/痤疮（78%）；在LUX-Lung 2研究^[9]^中，阿法替尼40 mg组有30例，最常见的不良反应为腹泻（97%）、皮疹/痤疮（90%）；99例入50 mg组，最常见的不良反应包括腹泻（94%）、皮疹/痤疮（94%）。LUX-Lung 3研究^[10]^中229例接受阿法替尼治疗，起始剂量为40 mg，结果表明腹泻（95%）、皮疹/痤疮（89%）是发生率最高的副反应，与本研究相同。LUX-Lung 4^[11]^研究入组62例，阿法替尼起始剂量为50 mg，所有患者（100%）均出现腹泻，皮疹/痤疮（92%）的发生率也较高。本研究中腹泻见于100%患者，皮疹见于80%患者，位列阿法替尼副反应发生率的前2位，与多家报道一致。

我们还观察到，本研究中阿法替尼起始剂量为40 mg，粘膜炎/口腔炎的发生率为80%，高于Yap^[7]^报道的40 mg组的23%（*n*=6）和LUX-Lung 2^[9]^中40 mg组的50%（*n*=15），与LUX-Lung 2^[9]^、LUX-Lung 4^[11]^中50 mg组的90%（*n*=89）、86%（*n*=53）较为接近。LUX-Lung 3^[10]^中排在第3位的不良反应是粘膜炎/口腔炎，占72%（*n*=165），这一结果与本研究结果相似。而在LUX-Lung 1^[8]^中，阿法替尼剂量是50 mg，发生口腔炎的比例为61%。本研究中粘膜炎/口腔炎的发生率高于同等剂量下的其他研究，考虑与本研究样本量较小有关。另外，除LUX-Lung 4外，其他研究中均存在一定比例的非亚裔患者（占13%-94%），尤其是Yap的Ⅰ期试验中非亚裔占94%（所有剂量组粘膜炎的发生率仅19%），而本研究中5例患者均为亚裔，提示人种的不同是否与阿法替尼副作用的不同有关尚需进一步研究。在上述几项研究^[7-9, 11]^中均有阿法替尼起始量为50 mg的人群，而本研究起始量为40 mg，依然出现较多的粘膜炎/口腔炎，提示需关注此项副反应。

关于不良反应的严重程度，本研究中阿法替尼的副反应程度较轻，均≤Ⅲ级，可耐受，能自行缓解或经治疗后减轻，与多项研究相似^[7, 9, 11]^。本研究中腹泻虽然发生率最高（100%），但其程度最轻（均为Ⅰ级），对阿法替尼的使用无影响，在LUX-Lung 2^[9]^中也有相似的结果。

从阿法替尼副反应的分布特点看，本研究患者主要集中在胃肠道、皮肤和皮下组织、给药部位异常与全身不适等方面，占全体不良事件的79%。除此之外，我们还观察到一些相对少见的副反应：如指甲改变（甲沟炎）、面部水肿、心悸。上述结果与许多学者的报道^[6]^一致。

我们观察了不良反应对用药剂量的影响。本研究中有3（60%）例经历了暂停药、减量，这一比例高于LUX-Lung 1^[8]^的38%（*n*=150）减量、18%（*n*=70）停药和LUX-Lung 2^[9]^40 mg组的37%（*n*=11）减量、LUX-Lung 3^[10]^8%的停药。相反，LUX-Lung 4^[11]^中减量者占69%（*n*=43），停药者占29%（*n*=18），高于本研究结果，考虑与起始剂量（50 mg）高有关。本研究中减量原因主要是粘膜炎/口腔炎，其次为皮肤异常（手足皮肤反应）。而其他研究的减量原因包括：皮肤改变（皮疹、痤疮）、腹泻、小肠炎、呕吐、呼吸衰竭、可疑的间质性肺病等^[9-11]^，因粘膜炎/口腔炎而减量的仅在LUX-Lung 4^[11]^中有2例（3%）报道。

关于药物相关的SAE，LUX-Lung 1^[8]^中发生率为10%（*n*=39），最常见的为腹泻17例（4%），药物相关性肾损害9例（2%）；LUX-Lung 2^[9]^中有12%（*n*=16）发生，包括皮疹或痤疮、蜂窝织炎、腹泻、乏力、间质性肺病等。LUX-Lung 3^[10]^中4例（2%）死亡考虑与治疗相关（2例呼吸失代偿、1例脓毒症、1例未知）。LUX-Lung 4^[11]^中有7例（11%）SAE，腹泻最为常见。本研究未观察到SAE，考虑与样本量较小有关。

阿法替尼作为第二代EGFR-TKIs，其不良事件频度与第一代EGFR-TKIs^[12-15]^有所不同。第一代EGFR-TKIs皮疹发生率最高，而第二代EGFR-TKIs腹泻与皮疹同为最常见的副反应，多数研究中腹泻发生率甚至略高于皮疹。两代EGFR-TKIs相比，转氨酶升高的发生率一代EGFR-TKIs高于二代，粘膜炎/口腔炎发生率二代EGFR-TKIs高于一代。另外，第一代EGFR-TKIs少见的副反应——间质性肺病^[3]^备受关注，LUX-Lung 2^[9]^中发生可疑的间质性肺病4例（3%），其中1例为致命性；LUX-Lung 3^[10]^中出现3例（1%）可能相关的间质性肺病样反应；LUX-Lung 4^[11]^中发生2例（3%），但其他多项研究未发现阿法替尼导致间质性肺病，本研究亦未发现此副反应。

LUX-Lung 6的最新研究^[16]^结果表明，阿法替尼最常见的副反应是皮疹/痤疮（15%）、腹泻（5%）、口腔炎/粘膜炎（5%），与上述多项研究结果类似，但其发生率远低于本研究及多项已公布的研究结果^[8-11]^。考虑这与各项研究的入组人数悬殊较大有关。

本研究共入组5例，4例可评价疗效，ORR 50%，3例获得PFS，中位PFS 9.7个月，与LUX-Lung 6中ORR 67%，中位PFS 11.0个月的研究结果相近，同样也与LUX-Lung 3^[10]^的中位PFS 11.1个月接近。LUX-Lung 6与LUX-Lung 3均是在*EGFR*突变阳性的NSCLC患者中进行一线治疗的临床试验，其结果具有可比性。3例获得PFS的患者中，2例进展后接受了化疗及靶向治疗，另1例拒绝后续治疗，中位OS 18.4个月，小于LUX-Lung 2的中位OS 24.8个月，考虑与两研究入组人数差异悬殊有关。本研究中2例患者分别在服药0.3个月、2.4个月后尚未进展的情况下退出研究，导致样本量较小，对PFS、OS造成一定影响。

综上所述，阿法替尼作为第二代EGFR-TKI的代表药物，用于具有*EGFR*活性突变的肺腺癌患者的一线治疗是肯定的。其最常见副反应为腹泻和皮疹，粘膜炎/口腔炎的发生率亦较高。由于本研究病例数仅5例，我们得到的只是一个初步的、不排除具有偏颇性的结论，此结论尚需更多的研究进一步证实。相信随着该药在中国临床研究的不断深入，我们对阿法替尼的副反应、作用机理、疗效等会有更加全面、深刻的认识。
